# Deep Learning based Radiomics (DLR) and its usage in noninvasive IDH1 prediction for low grade glioma

**DOI:** 10.1038/s41598-017-05848-2

**Published:** 2017-07-14

**Authors:** Zeju Li, Yuanyuan Wang, Jinhua Yu, Yi Guo, Wei Cao

**Affiliations:** 1 Department of Electronic Engineering, Fudan University, Shanghai, China; 2 Key laboratory of Medical Imaging Computing and Computer Assisted Intervention of Shanghai, Shanghai, China; 3 Department of micro-electronics, Fudan University, Shanghai, China

## Abstract

Deep learning-based radiomics (DLR) was developed to extract deep information from multiple modalities of magnetic resonance (MR) images. The performance of DLR for predicting the mutation status of isocitrate dehydrogenase 1 (IDH1) was validated in a dataset of 151 patients with low-grade glioma. A modified convolutional neural network (CNN) structure with 6 convolutional layers and a fully connected layer with 4096 neurons was used to segment tumors. Instead of calculating image features from segmented images, as typically performed for normal radiomics approaches, image features were obtained by normalizing the information of the last convolutional layers of the CNN. Fisher vector was used to encode the CNN features from image slices of different sizes. High-throughput features with dimensionality greater than 1.6*10^4^ were obtained from the CNN. Paired t-tests and F-scores were used to select CNN features that were able to discriminate IDH1. With the same dataset, the area under the operating characteristic curve (AUC) of the normal radiomics method was 86% for IDH1 estimation, whereas for DLR the AUC was 92%. The AUC of IDH1 estimation was further improved to 95% using DLR based on multiple-modality MR images. DLR could be a powerful way to extract deep information from medical images.

## Introduction

Radiomics is an emerging method that uses a series of qualitative and quantitative analyses of high-throughput image features to obtain predictive or prognostic information from medical images^1^. Recently, radiomics methods have been used to analyze various medical images and have provided information related to patient outcomes, tumor phenotypes and the gene-protein signatures of different diseases. For instance, Aerts *et al*. presented a radiomics method to decode tumor phenotypes of both lung cancer and head-and-neck cancer based on computed tomography (CT) data^2^. That study showed that it is possible to improve cancer diagnosis and treatment by using routinely collected medical images. The features described in that study were divided into four groups: intensity, shape, texture and wavelet features. Then, the features with the best performance in each group were combined to establish the radiomics model. Kumar *et al*. summarized and demonstrated the processes and challenges of radiomics^3^ with non-small-cell lung cancer (NSCLC). They proposed an NSCLC classification model that uses radiomics features. More recently, Vallieres *et al*. evaluated the lung metastasis risk of soft-tissue sarcomas (STSs) with a radiomics model^4^. Several non-textural features and texture features were extracted from the tumor regions in multiple medical scans. Their research showed that a combination of features extracted from positron emission computed tomography (PET) and magnetic resonance (MR) images could provide good metastasis estimation. Indeed, certain features in MR images were reported to exhibit characteristics associated with clinical diagnosis^5^. Gutman *et al*. analyzed the association between visual features of MR images and genetic alterations, gene expression and patient survival of glioblastoma (GBM)^6^. Visually Accessible Rembrandt Image (VASARI) features of the tumor regions from MR images were used in their study, and the results demonstrated a significant association between contrast-enhanced tumors and the Verhaak gene expression classification. A series of analyses of GBM was conducted by Gavaert *et al*.^7^. Gavaert *et al*. generated computational image features of necrosis, enhancements and edema regions of interests (ROIs). Their results showed that a certain image features correlated with molecular subgroups.

Normally, the process of radiomics analysis includes image acquisition, image segmentation, feature extraction, feature selection and informatics analyses^3^. There are three basic problems with existing radiomics sequencing methods. First, the image segmentation step usually relies on manual delineation. This process is both time-consuming and subject to inter- or intra-segmentation variation. Second, even if the image segmentation is accurate, there is no standard evaluation method for image feature extraction. Different image features will lead to different analysis results. Because it is difficult to verify the accuracy and reproducibility of image features, extra errors may be introduced due to the miscalculation of image features. Last, but not least, current radiomics methods often characterize medical images by using several groups of image features, including intensity, shape, texture and wavelets. Although many such image features can be calculated, it is not possible for all these imaging characteristics of segmented areas to be included in the predesigned features.

To overcome the shortcomings of radiomics methods, we developed a more advanced method, called deep learning-based radiomics (DLR). DLR obtains radiomics features by normalizing the information from a deep neural network designed for image segmentation. The main assumption of DLR is that once the image has been segmented accurately by the deep neural network, all the information about the segmented region will have already been installed in the network. Unlike current radiomics methods, in DLR, the high-throughput image features are directly extracted from the deep neural network. Because DLR does not involve extra feature extraction operations, no extra errors will be introduced into the radiomics analysis because of feature calculations. The effectiveness of features is related only to the quality of segmentation. If the tumor has been segmented precisely, the accuracy and effectiveness of the image features can be guaranteed.

In the proposed DLR, a convolutional neural network (CNN) is used. CNN is a representative method used for deep learning, and it has been successfully applied to the field of image segmentation^8^. Recently, many groups have used CNN for the segmentation of medical images, and it has provided better results than traditional methods^9^. In glioma segmentation based on MR images, most of the CNN methods were proposed for high-grade gliomas^10, 11^. Compared with high-grade gliomas, low-grade gliomas are smaller and have lower contrast with the surrounding tissues^12^. Existing CNN structures would not work well for segmentation of low-grade gliomas. A major architecture adjustment of CNN is therefore essential for both image segmentation and feature extraction. To address the challenging characteristics of low-grade gliomas, we used a modified CNN architecture with 6 convolutional layers and a fully connected layer with 4096 neurons for segmentation.

With the more accurate segmentation results obtained by CNN, more information can be extracted. Unlike traditional calculated features, CNN features preserve a great deal of the global spatial information using the operations of convolutional kernels for the entire image^13^. Indeed, CNN features have shown better performance than traditionally calculated features in many domains, such as scene recognition, domain adaptation and fine-grained recognition^14^. More recently, the features in a CNN showed promising results for texture attribute recognition, and CNN outperformed traditional approaches by more than 10%^15^. In DLR, CNN features are extracted from the last convolutional layer. A Fisher vector is used to normalize the network information from MR imaging slices of different sizes; 16,384 high-throughput image features were generated from the CNN for each case.

The performance of the proposed DLR was validated by using it to predict the isocitrate dehydrogenase 1 (IDH1) statuses of low-grade gliomas^16, 17^. Since the introduction of the concept of molecular diagnosis for glioma, which is the most common malignant brain tumor, large-scale genomics data are now available^18–20^. In the latest version of the WHO 2016^21^, molecular diagnosis and pathological diagnosis were integrated for central nervous system tumors, including glioma. Among all the molecular biomarkers, the IDH1 gene is the most important because of its unique diagnostic and predictive value. IDH1 mutation status accounts for more than 50% of the predictive value in low-grade glioma^22^. The treatment regimen varies greatly in low-grade glioma according to IDH1 status^23^. Therefore, accurate prediction of IDH1 mutation status via noninvasive methods has been widely explored. Here, we used DLR to determine IDH1 mutation status in a low-grade glioma cohort composed of 151 patients. We demonstrate that DLR is a useful and accurate tool for predicting IDH1 mutation status in low-grade gliomas.

## Results

### Participants

A cohort of 151 cases was selected from the image bank of the Department of Neurosurgery, Huashan Hospital. All patients enrolled in this study were diagnosed with grade II glioma. The diagnoses of low-grade glioma were re-confirmed independently by 2 pathologists for each case, and the IDH1 mutation statuses were confirmed by Sanger sequencing. Detailed information about the enrolled patients is summarized in Table 1. The cases were divided into two cohorts. The first cohort, consisting of 119 cases with both T2 flair and T1 contrast images, was used to test the DLR performance based on multiple MR imaging modalities. The second cohort, consisting of 110 cases with T2 flair images, was used to compare the IDH1 prediction performance between DLR and the normal radiomics approach. The second cohort was used in our previous radiomics study for the same purpose^24^.

**Table 1:** Characteristics of patients in all datasets, first cohort and second cohort.

Parameters	Total Cases	IDH1 Mutation Status		p-value
		Mutation	Wild Type	
***All the dataset***				
Number of samples	151	112	39	
**Sex**				
Male	81(54.6%)	58(51.8%)	23(59.0%)	0.44
Female	70(46.4%)	54(48.2%)	16(41.0%)	
**Age (years)**				
Mean ± standard deviation	40.7 ± 10.8	38.7 ± 10.7	43.5 ± 12.1	0.03
**Tumor volume (cubic centimeters)**				
Mean ± standard deviation	68.1 ± 47.4	69.0 ± 48.0	65.8 ± 46.8	0.843
**Histopathological diagnosis**				
Astrocytoma	81(54.6%)	54(48.2%)	27(39.2%)	0.01
Oligodendroglioma	31(20.5%)	27(24.1%)	4(10.2%)	
Oligoastrocytoma	39(25.8%)	31(27.7%)	8(20.5%)	
***First cohort***				
Number of samples	119	89	30	
**Sex**				
Male	67(56.3%)	48(54.0%)	19(63.3%)	0.37
Female	52(43.7%)	41(46.1%)	11(36.7%)	
**Age (years)**				
Mean ± standard deviation	39.6 ± 10.2	37.9 ± 8.9	44.2 ± 11.7	0.03
**Tumor volume (cubic centimeters)**				
Mean ± standard deviation	68.3 ± 57.4	66.7 ± 55.6	73.1 ± 63.0	0.146
**Histopathological diagnosis**				
Astrocytoma	69(58.0%)	47(52.8%)	22(73.3%)	0.03
Oligodendroglioma	24(20.2%)	21(23.6%)	3(10.0%)	
Oligoastrocytoma	26(21.8%)	21(23.6%)	5(16.7%)	
**Diagnosis time**				
Before 2015	85(71.4%)	63(70.8%)	22(73.3%)	0.79
After 2015	34(28.6%)	26(29.2%)	8(26.7%)	
***Second cohort***				
Number of samples	110	76	34	
**Sex**				
Male	54(49.1%)	33(43.4%)	21(61.7%)	0.08
Female	56(50.9%)	43(56.6%)	13(38.2%)	
**Age (years)**				
Mean ± standard deviation	40.3 ± 11.3	39.0 ± 10.7	43.4 ± 12.7	0.06
**Tumor volume (cubic centimeters)**				
Mean ± standard deviation	68.1 ± 57.9	74.3 ± 62.5	54.3 ± 43.8	0.06
**Histopathological diagnosis**				
Astrocytoma	55(50.0%)	31(40.8%)	24(70.6%)﻿	0.01
Oligodendroglioma	21(19.1%)	18(23.7%)	3(8.8%)	
Oligoastrocytoma	34(30.9%)	27(35.5%)	7(20.6%)	

### Tumor segmentation results

**Figure 1:**
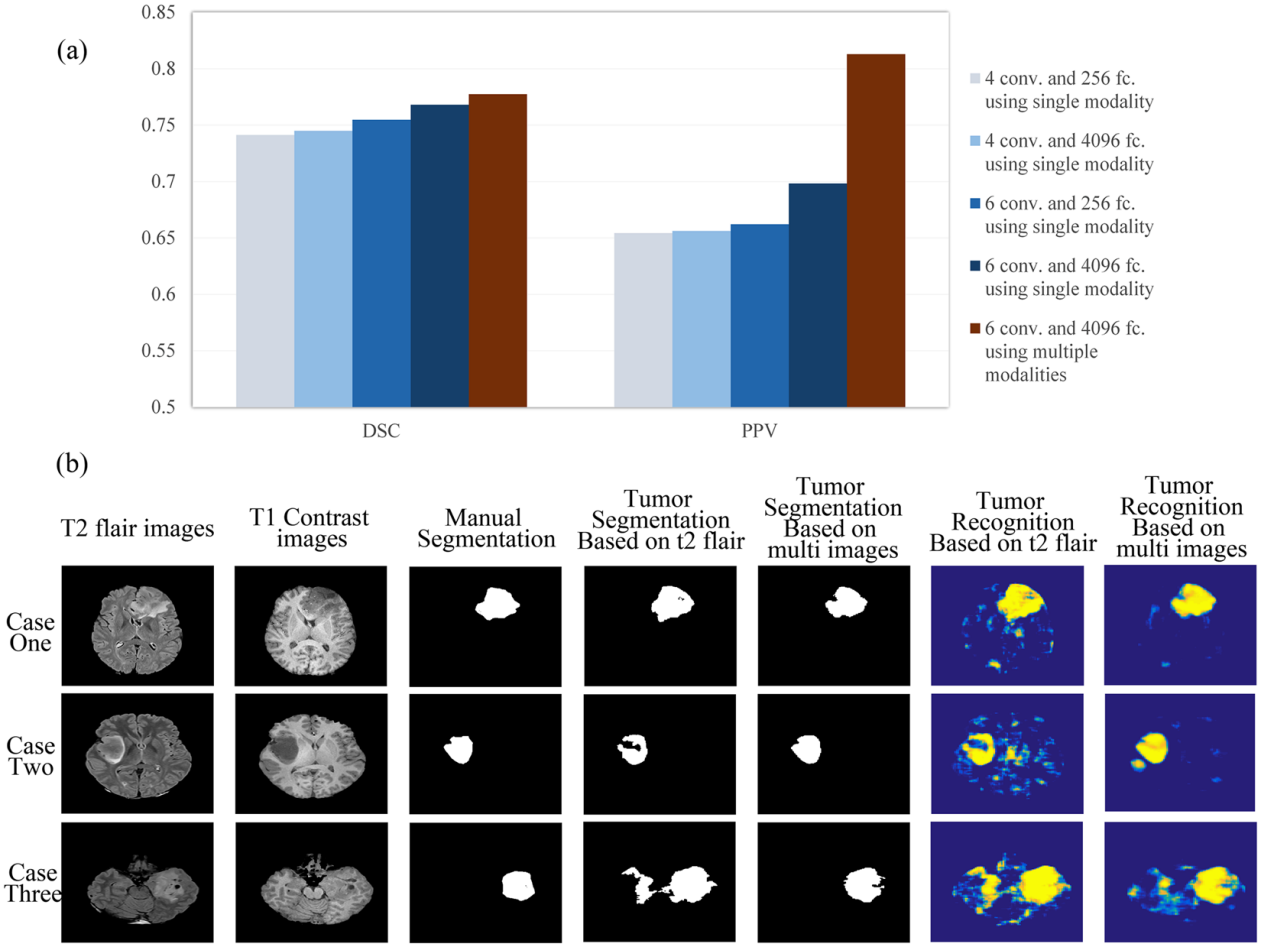
Tumor segmentation results for single-modal images and multi-modal images using different network structures. (**a**) Comparison between the indexes of different segmentation results of different CNNs. Conv. indicates the number of convolutional layers in the CNN structures, and fc. indicates the number of neurons in the fully connected layers in the CNN structures. (**b**) Three typical cases with segmentation results for the CNN with 6 convolutional layers and fully connected layers with 4096 neurons.

The tumor segmentation results are shown in Fig. 1. The evaluation indexes are explained in the Supplementary methods section, and the results of different CNN structures are presented in Supplementary Table 1. As expected, the network was able to segment the tumor regions.

The results in Fig. 1(a) show that increasing the network depth or increasing the number of neurons in the fully connected layers led to more precise segmentation results. Ultimately, using a deeper network structure and employing more neurons in the fully connected layers at the same time led to the best overall segmentation results. Further performance improvements were achieved by combining multiple modal MR images.

We present three typical segmentation results of CNNs trained with single-modality and multi-modality images in Fig. 1(b). As shown in the pictures, the CNN provided correct segmentation results for the tumor regions. The false recognition of other brain structures was reduced (in cases one and three), and the tumor region was recognized more accurately (in case two).

### Prediction results for IDH1 using DLR

To better understand the features extracted by DLR, we analyzed the selected features from multiple MR imaging modalities. In general, the CNN features represented the characteristics of the responses of deep filter banks in the last convolutional layer. The dimension of CNN features was normalized using their distribution statistics with regard to 64 Gaussian kernels. Detailed information of the selected CNN features is presented in Supplementary Figure 1. The majority of the features were only related to a few Gaussian kernels. In other words, there are many correspondences among the features that showed significant responses to IDH1 status. On the other side, several filter banks show great responses to IDH1 status.

**Figure 2:**
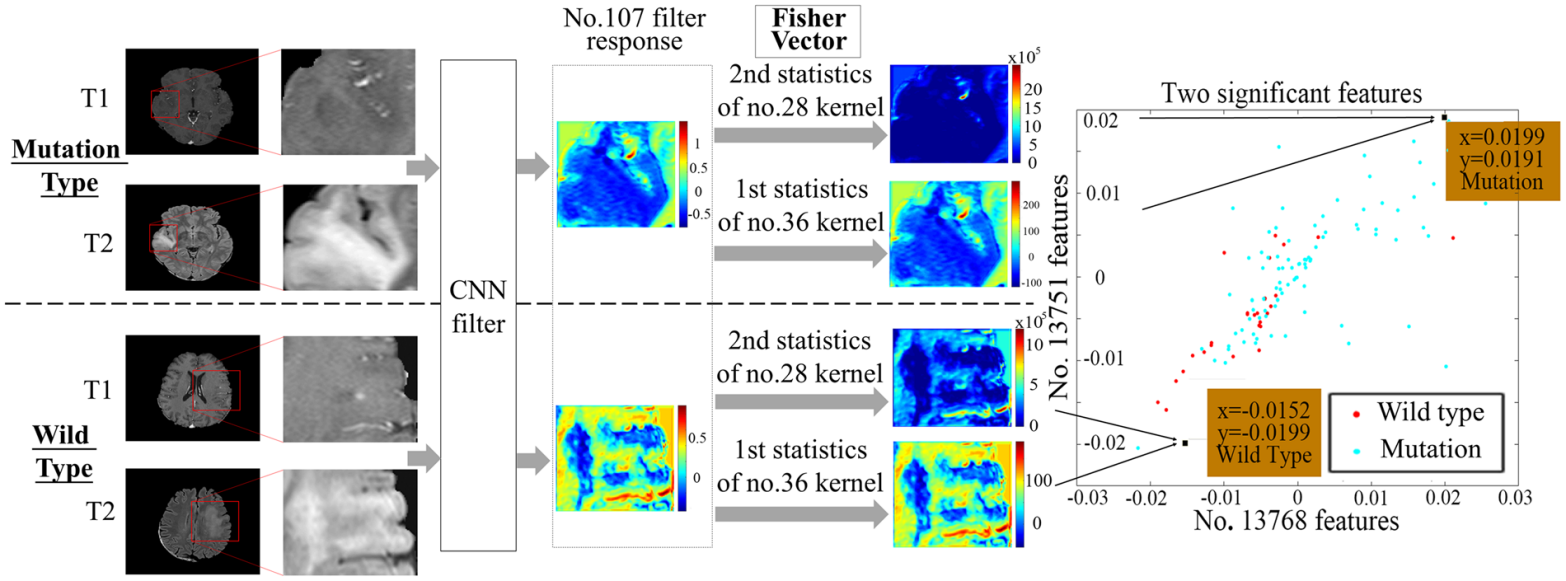
CNN features from the last convolutional layers. (**a**) An example of specific CNN features. Deep filter responses showed noticeable differences between wild-type and mutant IDH1, and the Fisher vectors could successfully represent the differences.

An example that illustrates our method is shown in Fig. 2. Two typical IDH1 mutation and wild-type cases were used as examples. The ROIs were entered into the network, and CNN features from the last convolutional layers were extracted and encoded by a Fisher vector. Of all the 16,384 CNN features, no. 13,751 and no. 13,768 were the two most significant in their response to IDH1 status (with F-scores of 0.3501 and 0.3561, respectively). The two features corresponded to the first order statistics of the no. 36 kernel of the no. 107 deep filter response and the second order statistics of the no. 28 kernel of the no. 107 deep filter response. We visualized the feature maps of the no. 107 filter for the two cases in Fig. 2. Surprisingly, although the inputs of the two types of tumor were similar, the outputs from the no. 107 filter response were almost entirely different.

Next, the first order statistics of the no. 36 kernel and the second order statistics of the no. 28 kernel were calculated; these refer to the designs of the no. 13,751 and no. 13,768 features. The characteristics of the filter response were reflected in the two parameters. As the second order statistics of the no. 28 kernel show, the no. 107 filter response of the wild-type tumor was more internally complicated and had more texture information. As the first order statistics of the no. 36 kernel show, the no. 107 filter response of the mutation-bearing tumors had lower intensity and was more gathered around the no. 36 kernel (the no. 36 kernel had a mean value of −0.1352 and a variance of 0.0055). Thus, the prediction based on CNN features clearly provided good results. Indeed, the two types of tumors have significantly different responses in deep filter banks.

To make the analysis more comprehensive, we show the feature maps from the no. 107 filter in Supplementary Figure 2. The feature maps of IDH1 mutant gliomas showed a more uniform distribution. By contrast, the internal textures of the feature maps of the wild-type gliomas were more complex, and the response regions were more irregular. However, these differences were not obvious in the original MR images.

### Comparison with normal radiomics methods

**Figure 3:**
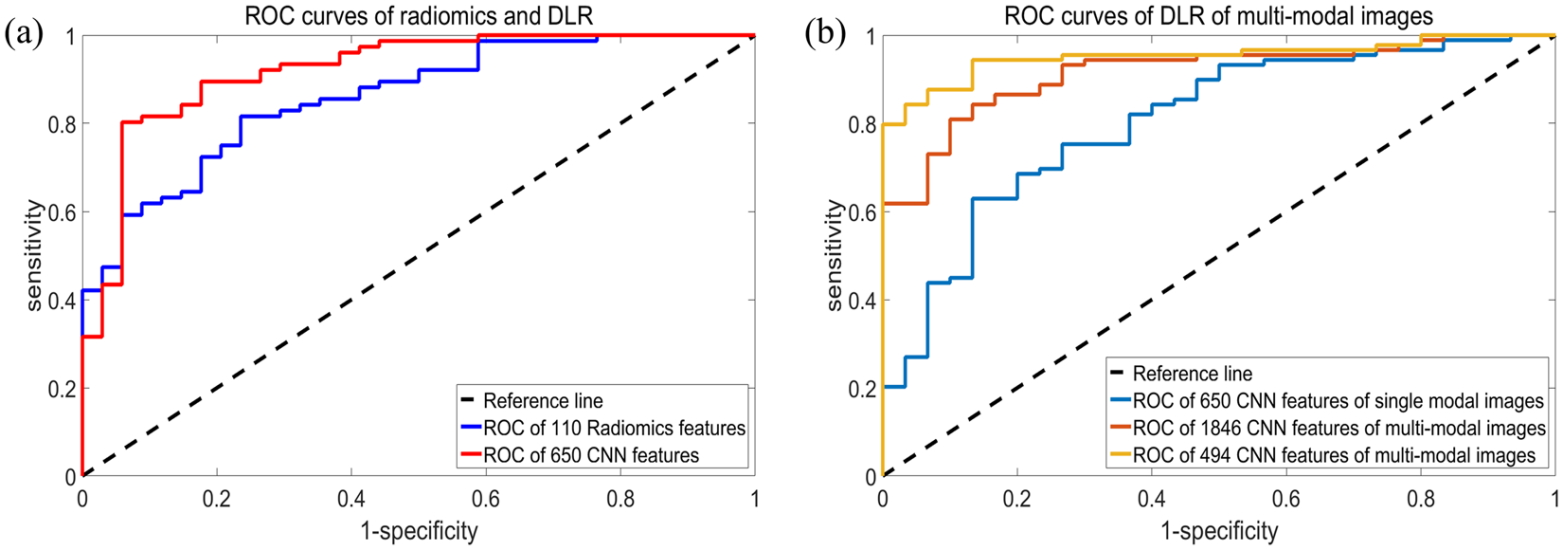
ROC curves of the prediction results. (**a**) ROC curves of the radiomics features and DLR of the second cohort with single-modal images. (**b**) ROC curves of DLR of the first cohort with multiple modal images are shown on the right.

In our previous study^24^, we used normal radiomics based on calculated features to estimate the IDH1 status of the second cohort used in this current study. In this study^24^, we extracted 671 image features from the segmented images. To evaluate the CNN features in the radiomics analysis, we used the same cohort and analysis process, but we replaced the 671 features with 16,384 CNN features. The evaluation parameters of the prediction results are shown in Table 2(a). A comparison of the receiver operating characteristic (ROC) curves is presented in Fig. 3(a). The features used are summarized in Supplementary Table 2. The results show a great improvement as a result of using the CNN features.

**Table 2:** Prediction results of different cohorts using different methods.

Dataset	Methods	AUC	ACC	SENS	SPEC	PPV	NPV	MCC
**(a) Normal Radiomics vs. DLR (single modality: T2 flair)**								
Second cohort with leave-one-out cross-validation	Radiomics^24^	0.8572	0.8000	0.8289	**0.7353**	0.8750	0.6579	0.5483
	DLR	**0.9207**	**0.8636**	**0.9342**	0.7059	**0.8765**	**0.8276**	**0.6713**
**(b) DLR based on single modality vs. multiple modality**								
First cohort with leave-one-out cross-validation	DLR with single modality	0.8045	0.8235	0.9326	0.5000	0.8469	0.7143	0.4927
	DLR with multiple modality	0.9157	0.8655	**0.9438**	0.6333	0.8842	0.7917	0.6246
	DLR with multiple modality improved by further feature selection	**0.9521**	**0.9244**	**0.9438**	**0.8667**	**0.9545**	**0.8387**	**0.8018**
**(C) Divided test set based on diagnosis time**								
First cohort with divided test set	DLR with multiple modality	0.9615	0.9118	0.9231	0.8750	0.9600	0.7778	0.7673

(a) Comparison of IDH1 prediction results between radiomics and DLR using T2 flair modal MR images of the first cohort by leave-one-out cross-validation SVM. (b) Prediction results of IDH1 using multi-modal MR images of the second cohort by leave-one-out cross-validation SVM and (c) validation on a divided test set. The data set was divided according to the diagnosis time.

The modified CNN architecture was able to provide joint information of multiple modalities for the DLR analysis. The performance of multiple modalities was evaluated using the dataset of the first cohort with both T2 flair images and T1 contrast images. Two evaluation methods were used: leave-one-out cross-validation and validation based on time of diagnosis. The evaluation parameters of the prediction results based on leave-one-out cross-validation are shown in Table 2(b), and a comparison of the ROC curves is presented in Fig. 3(b).

The results showed that DLR provided better prediction results by using multi-modal images rather than single-modal images. Moreover, further feature selection would improve the predictive ability of this model. The performance of further feature selection is shown in Supplementary Figure 3.

Another experiment was carried out to validate our method. Eighty-five patients diagnosed before 2015 (63 mutations and 22 wild type) were used as a training set, and 34 patients diagnosed after 2015 (26 mutations and 8 wild type) were used as the validation set. The same features of multi-modal DLR with further feature selection were used for IDH1 status prediction. The results were similar to those obtained with leave-one-out cross-validation, as shown in Table 2(c).

### DLR performance with CNN features with different layers

As shown in Fig. 1, the CNN architecture that we designed demonstrated better performance than previous methods for tumor segmentation. We demonstrated the importance of deepening the network structure by comparing prediction results using CNN features from different layers. The prediction results are summarized in Table 3. The numbers of features used for prediction in each case are summarized in Supplementary Table 2. As shown in the table, the overall prediction results improved when the network was built deeper. However, when we proceeded to the fully connected layers, no further improvement was achieved.

**Table 3:** Comparison of IDH1 prediction results using CNN features from different layers.

Methods	AUC	ACC	SENS	SPEC	PPV	NPV	MCC
Conv.1	0.6165	0.5630	0.5393	0.6333	0.8136	0.3167	0.1499
Conv.2	0.7109	0.6387	0.6404	0.6333	0.8382	0.3725	0.2402
Conv.3	0.8858	0.8403	0.9213	0.6000	0.8723	0.7200	0.5557
Conv.4	0.8734	0.7899	0.8876	0.5000	0.8404	0.6000	0.4132
Conv.5	0.9004	0.8571	0.9101	**0.7000**	**0.9000**	0.7241	0.6171
Fc.7	0.8614	0.8319	0.9326	0.5333	0.8557	0.7273	0.5212
Fc.8	0.7524	0.7647	0.8876	0.4000	0.8144	0.5455	0.3217
Conv.6	**0.9157**	**0.8655**	**0.9438**	0.6333	0.8842	**0.7917**	**0.6246**

Conv. means the convolutional layers, and fc. represents the fully connected layers. The same post-processing was applied in each situation.

**Figure 4:**
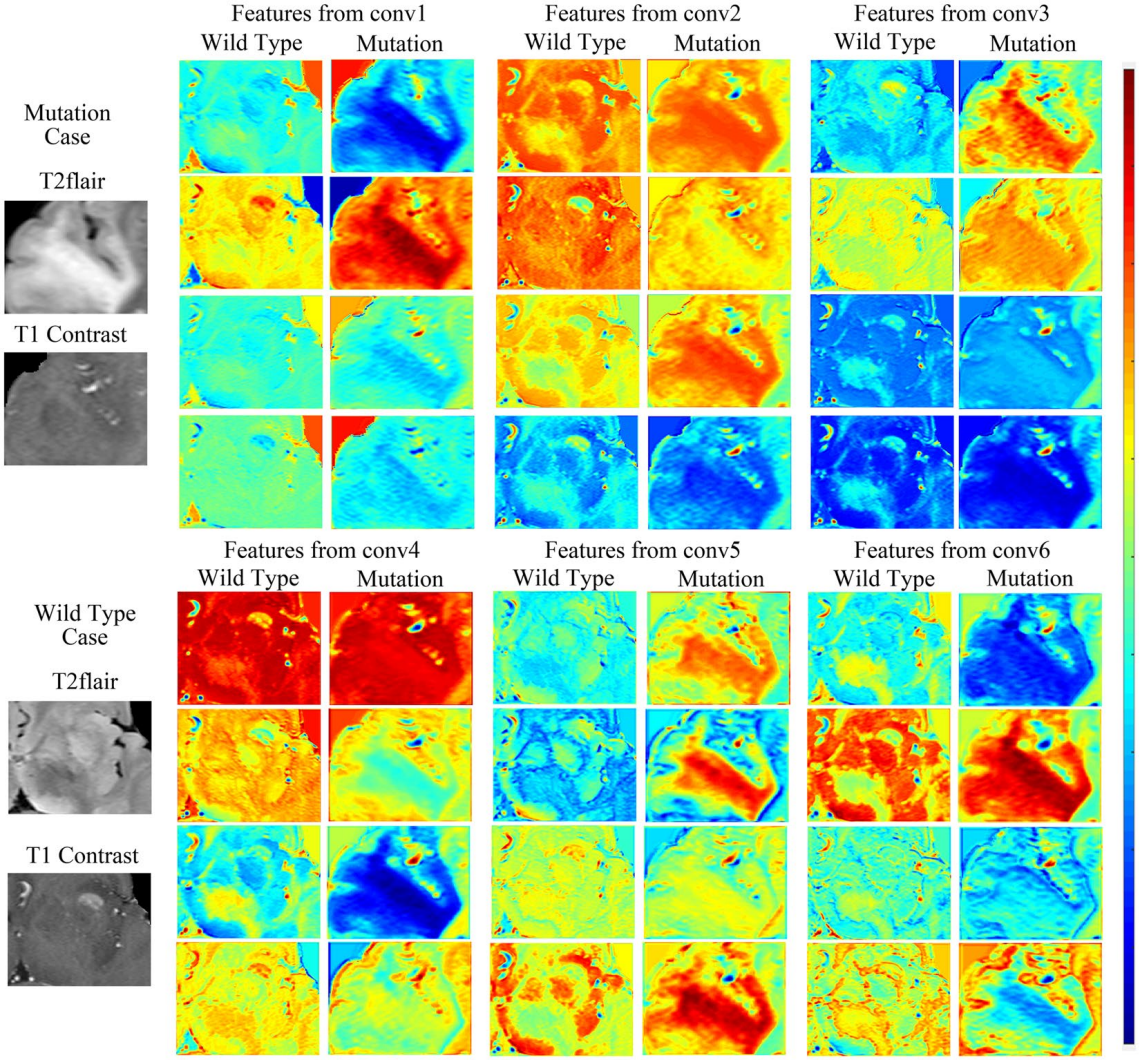
Feature maps of CNN features from different convolutional layers. The four most significant filter banks of different layers were selected. As shown in the figures, feature maps of deeper layers represented more detailed characteristics.

Next, we illustrated the importance of building a high-performance CNN with feature maps obtained from different depths of the network. Features maps were the output planes of different deep convolutional kernels and could be thought of as the responses of the local feature extractors. Feature maps of the four most significant filter banks of each convolutional layer are presented in Fig. 4. These feature maps are the responses of the CNN to the same two cases. Normally, feature maps are thought to provide more detailed information as the network becomes deeper^13^. In the lower convolutional layers, the CNN only acquires intensity and shape information from the tumor, and most filter banks do not show distinct responses to different disease phenotypes. As the CNN structure becomes deeper and the number of network parameters increases, the features become more difficult to describe. However, we can see from the figure that the feature maps of the deeper layers have more information about the details and internal textures. The mutation cases and wild-type cases can be distinguished in various ways. To demonstrate the ability of DLR to differentiate between phenotypes, we present a boxplot of the ten most significant features in Supplementary Figure 4.

### Operation time

All of our computations were carried out using an NVIDIA Quadro 600 GPU on an Intel Xeon E5620 2.40 GHz machine. It took 38 hours to train the tumor recognition CNN. Eighteen minutes were required per case for the tumor segmentation, and 40–80 seconds per case were needed for CNN feature extraction, depending on the tumor size. Approximately 2 seconds were used for the encoding of the Fisher vector.

## Discussion

A modified CNN architecture designed for low-grade glioma segmentation was introduced in this study. Compared to previous neural networks, the proposed CNN was built with more convolutional layers and more neurons in the fully connected layers. Increasing the depth of the network and adding parameters enabled the CNN to make more adjustments to the input images and improved the learning ability of the CNN. For these reasons, we were able to obtain more accurate segmentation results and better tumor identification using the CNN. Similar to CNN methods that process natural images in three-channel RGB^25^, we used multi-modal MR images as inputs for the CNN. CNN structures based on multiple imaging modalities obtained more information. More image information helped the CNN to better detect the tumor regions. Therefore, incorrect segmentation for non-tumor regions was reduced, and the identification of tumor regions was precise.

Radiomics has become a popular method to extract prognostic information from medical images that is not visible to the human eye. Many radiomics features can be analyzed using medical images. As topical issues, radiomics methods have already been widely adopted for the noninvasive analysis of genetic and clinical information in different medical fields. The success of radiomics is based on the idea that medical images can provide much information about the internal state, which could be related to disease characteristics and may help in treating and understanding disease^26^. Radiomics can be used to obtain information by extracting a tremendous number of features from lesion areas. Although radiomics methods have made substantial progress, the features were designed without a special purpose in mind. The features used for different diseases are similar, and fewer than hundreds of features can be used, including intensity, shape, texture, wavelets and other descriptive features. The clinical characteristics may not be obvious in the images, and it is difficult and time-consuming to design a suite of related features. However, it is clearly difficult to tell whether the modeled radiomics features are related to a given problem. Traditional radiomics methods cannot guarantee that the features describe the pattern completely, and they cannot guarantee that the selected features are the best features.

The CNN features used in this study, unlike the features used in normal radiomics methods, were extracted directly from the networks. The CNNs were specifically trained on the given training data, and they were able to segment the tumor region automatically. The outstanding tumor recognition ability of the CNN inspired us to test whether more information about the disease could be extracted from the CNN structures. Indeed, we showed that the CNN features could better predict IDH1 status. We can summarize the superiority of DLR in three main improvements. First, the DLR can provide automatic segmentation results based on multi-modal images, and it can avoid the additional errors caused by subsequent feature extraction. We succeeded in simplifying the radiomics process into a more elegant procedure by directly extracting features from the network and avoiding difficulties in designing features. The process of DLR, including lesion area segmentation, can thus be fully automatic. This automation makes the DLR more robust and useful for the prediction of disease phenotypes. Second, the high-performance object recognition of the CNN was utilized. DLR can make full use of the image information within and near the tumor regions. CNN features were extracted directly from the network, and the most distinct deep filter responses were found. The strong connection between the deep information and IDH1 statuses benefited from the outstanding tumor recognition ability of the CNN. CNN structures with stronger tumor recognition ability, such as CNN structures based on multiple imaging modalities, could provide more value relevance information and lead to better discrimination of IDH1 statues. Third, the CNN can be specifically designed for the problem at hand, which means that CNN features can provide unique characteristics for particular images. The network was trained using medical images that needed to be processed. Therefore, the CNN learned a large number of general and unique features from the datasets, which would be impossible when using radiomics features or manually specified features.

In this study, information was extracted from the last convolutional layers. We were inspired to do this based on the idea that information from the deeper layers is more robust. The changes in details, such as shifting and scaling in the input images, would have an effect on the feature maps of lower layers but not on the deep layers^13^. This effect was illustrated by an experiment in this study. On the one hand, the nuanced differences between categories were highlighted by the abstract feature maps from the deep layers. These features were hard to depict, and they might correspond to the differences between disease phenotypes that are not visible to the human eye. They could help the classifier obtain more accurate prediction results. On the other hand, discrimination did not evolve any more by extracting features from the fully connected layers. The reason for this result is because the CNN features were too compressed in the fully connected layers, and the partial information reflecting the characteristics of disease phenotypes was lost.

Despite the diagnostic significance of glioma according to the new version the WHO criterion, the IDH1 mutation status was used to tailor personalized treatment regimens, including surgical extent and chemo-sensitivity. Patients with IDH1 mutations tend to have a positive prognosis. Currently, the prognosis evaluation of glioma is mainly decided by extensive histologic and genetic evaluation. Preoperative noninvasive prediction of IDH1 status could help guide treatment decisions in some cases, but it remains a challenge. Several noninvasive methods to predict IDH1 mutation status have been explored and reported during the last few years. Of these methods, MR imaging-oriented computational analysis is considered the most convenient and cost-effective. Two major methods have been proposed based on routine MR imaging or magnetic resonance spectroscopy (MRS). The first method is called image analysis, and it is based on routine clinical MR imaging scans. Yamashita *et al*. reported that tumor necrosis area and tumor blood flow are useful for predicting IDH1 mutation status^27^. A total of 66 patients with GBM were included in their study. IDH1 prediction results with area under the operating characteristic curve (AUC) values of 87.3% and 77.2% were obtained using the features of relative tumor blood flow and necrosis area, respectively. However, their method lacks an overall evaluation of the tumor regions, and the specific cutoff parameters in their research might be not applicable to other datasets. Recently, radiomics introduced another approach for the noninvasive prediction of IDH1 status. Our group also examined the possibility of predicting IDH1 mutation status using a radiomics method^24^. We carried out a preliminary classification prediction of IDH1 mutation statuses with T2 flair images of 110 patients with low-grade gliomas. Using 671 radiomics features describing the intensity, shape, texture and wavelet characteristics of the tumor regions, we obtained an accuracy of 80% and an AUC of 86%. Compared to IDH1 prediction methods based on image analysis, DLR combines the steps of image segmentation and feature extraction, and it efficiently avoids error propagation and could provide features with more comprehensive representation of the tumor regions that are relevant to the tumor molecular phenotype. Therefore, DLR outperforms other methods for IDH1 mutation prediction. Alternatively, the second method involves detecting metabolic changes caused by IDH1 mutation using MRS technology. When a tumor harbors an IDH1 mutation, it produces 2-hydroxyglutarate (2-HG), which is reflected in the MRS results^18, 28^. Several studies have reported ways to detect 2-HG by MRS using dedicated research scanners under certain conditions^29, 30^. Verma *et al*. developed two-dimensional localized correlated spectroscopy (2D L-COSY) at 7 tesla for the detection of 2-HG30. Nine patients were enrolled in their study. The results showed that 2-HG was detected in the IDH1-mutant gliomas but was absent in IDH1 wild-type gliomas. Lombardi *et al*. predicted the presence of IDH1 mutations based on 2-HG concentrations in the plasma and urine of 84 patients^31^. These methods provided an accuracy of 70%, a sensitivity of 63% and a specificity of 76% when the cutoff ratio of 2-HG was set to 19. De la Fuente *et al*. developed an MR imaging protocol to integrate 2-HG-MRS into routine imaging and validated the performance using 89 patients32. The results showed that 2-HG is closely linked to MRS voxel volume, with sensitivity values ranging from 8% for small tumors (<3.4 mL) to 91% for larger tumors (>8 mL). The main drawback of these 2-HG-based methods is that there are too many requirements for image acquisition, making it difficult to use in routine MR imaging processes^32^. Compared to IDH1 prediction methods based on measuring 2-HG, DLR can provide better results and relies only on the routine MR imaging modality. Our method can be applied to routinely collected MR imaging data instead of requiring additional samples or specific imaging parameters. Thus, it may be more practical to implement and more economical. One possible shortcoming of DLR may be encountered when processing data from different medical centers or data collected using different machines. Our CNN was built using MR images collected with the same parameters, but images with different imaging parameters might not be accurately identified by the settled network. This problem could be overcome by image normalization or fine-tuning using the new dataset.

In medical imaging applications, our method is able to noninvasively predict IDH1 mutation status with high accuracy by using routinely collected MR image modalities. In the future, we anticipate that DLR can be widely used in other cases where radiomics has been applied. Noninvasive prediction of other important glioma biomarkers, such as 1p19q and TERT, will be considered for future work. Prediction of survival time for GBM is also being considered as a future application of DLR. Our method can help related research obtain more specified features and greatly improve performance.

## Methods

### MR image data acquisition

All images were acquired using a 3 Tesla Siemens Trio scanner (Siemens, Erlangen, Germany). 3D T2 flair images were acquired using the following scan parameters: TR = 9000 ms, TE = 99 ms, TI = 2501 ms, flip angle = 150°, slice thickness = 2 mm and pixel spacing = 0.4688 mm. In addition, a high resolution, T1 contrast 3D images were acquired (TR = 1900 ms, TE = 2.93 ms, TI = 900 ms, flip angle = 9°, slice thickness = 1 mm and pixel spacing = 0.4883 mm). This experiment was approved by the Ethics Committee of Huashan Hospital, and informed consent was obtained from every patient. All experiments were carried out in accordance with relevant guidelines and regulations.

For each case, polymerase chain reaction (PCR) and subsequent sequencing analysis were performed as described previously^33^.

### DLR: Deep Learning-based Radiomics

**Figure 5:**
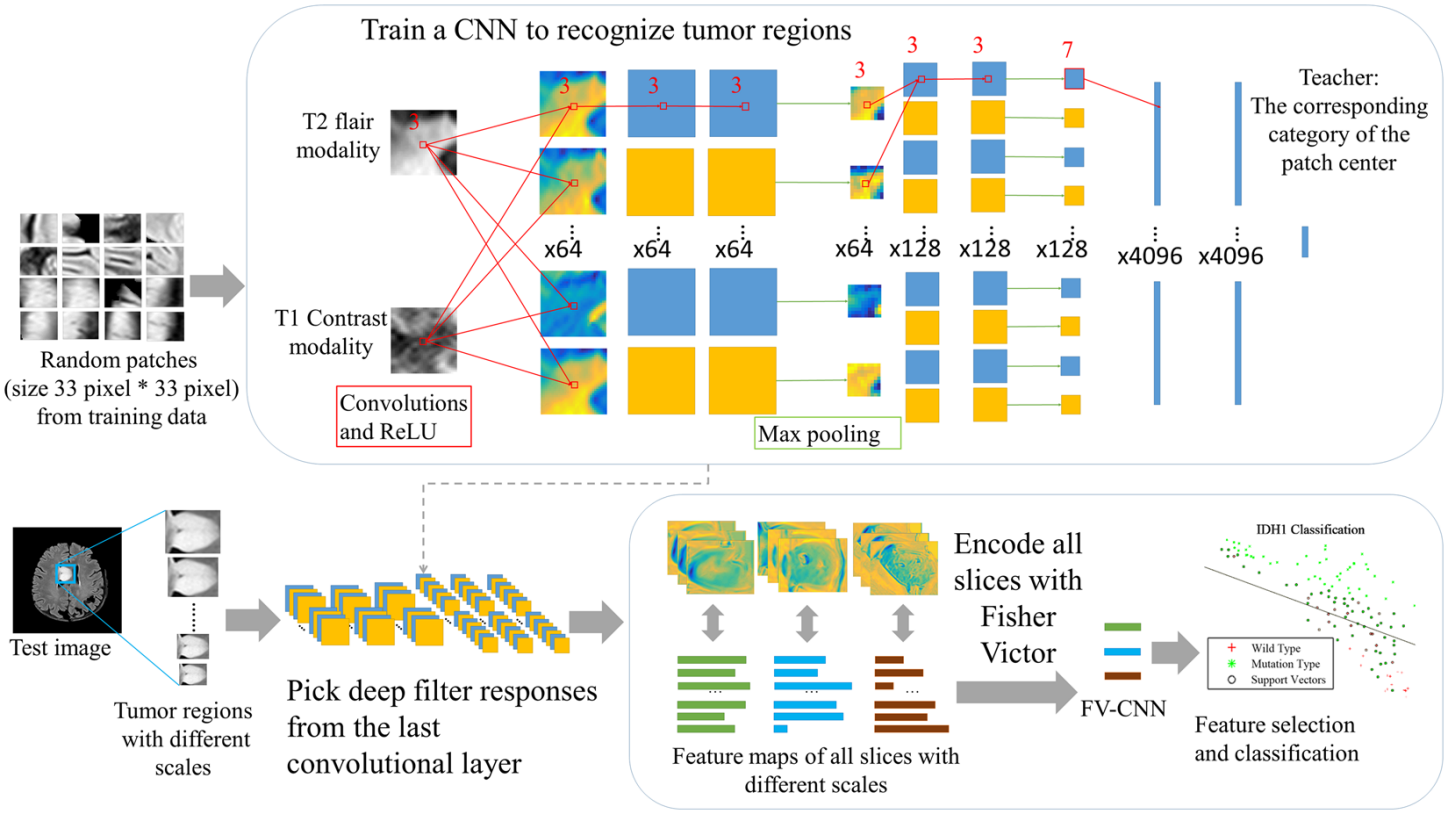
An overview of our DLR. Our approach included two selection steps. The first step is to recognize the tumor regions in the MR images based on a state-of-the-art CNN structure. In the second step, deep filter responses were extracted from the last convolutional layer through Fisher vector encoding. Then, the prediction results were evaluated by a leave-one-out cross-validation SVM.

An overview of DLR is shown in Fig. 5. Important concepts are described later.

### Data pre-processing

All T1 contrast MR images were first registered to the T2 flair MR images using Statistical Parametric Mapping (SPM)^34^. Then, Brainsuite^35^ was used to remove the skull and scalp from the brain MR images and correct the bias field for the MR images, leading to better recognition for the CNN. After that, the tumor regions were manually labeled by two experienced neurosurgeons. The manual segmentation results were used as ground truth in the CNN training phase.

### Tumor segmentation by deep learning

The structure of the CNN was mainly based on a previous study^10^. The selected method was used to participate in the Brain Tumor Segmentation Challenge (BRATS)^12^ and ranked first place and second place in BRATS 2013 and BRATS 2015, respectively.

The task of tumor region recognition was transformed into pixel-wise classification in the fashion of CNN. Taking into account the lack of brain MR image resolution in the coronal and sagittal planes, we considered using two-dimensional information for tumor recognition from the axial view. During the training phase, 33 × 33 pixel patches were extracted from the MR images. The mean gray levels of the patches in one channel were removed, and the gray values and variance were normalized. Normalized patches were input into the network with the category of the center points for training. However, the proportion of tumors in the images was very small. To better identify the tumor region, we used an unbalanced selection strategy, such that approximately 40% of the patches in the input contained tumor regions.

The convolutional kernels were convolved over the image to obtain the local features and global features of the images at the same time. The weights of the convolutional kernels were adapted by back-propagation in every training process. Therefore, the networks were adjusted to the characteristics of the input data. Stochastic gradient descent (SGD) was used as the parameter back-propagation algorithm.

When applying the structure to our dataset, we built it deeper and included more neurons in the fully connected layers. Detailed information about the structure used in our study is shown in Fig. 5 and Supplementary Table 3. Two convolutional layers were added, and the parameters in the fully connected layers were increased from 256 to 4096 to obtain more precise segmentation results. Rectified linear units (ReLU) were chosen as the activation function, and they were set following every convolutional layer. Additionally, dropout layers were applied after every fully connected layer. To ensure the accuracy and effectiveness of the image features, we selected network structures with 6 convolutional kernels and fully connected layers with 4096 neurons for subsequent processing.

Typically, full images were used as direct input to the network to obtain the segmentation results in the test phase. The same pre-processing parameters from the training phase were used, including the mean gray value, normalized gray value and variance. Bicubic interpolation was used for the up-sampling of network output to make up for the dimensional changes produced by the pooling process. After obtaining the segmentation results of the CNN output, we corrected the segmentation results by post-processing using several morphology methods. The largest connectivity region of each slice was first chosen as the candidate region. Then, the selected tumor regions were smoothed by a box filter with a 3-dimensional convolution kernel.

### Selection of deep filter responses

After confirming that the network was able to identify tumor regions, we added tumor region images to the network. To fully examine the tumor responses in the network, we used 10 images with different scales for each tumor slice, and the scale ratios ranged from 0.5 to 2.

The CNN was taken as an image filter, and the features of the tumor regions were generated from the feature maps from the last convolutional layer. However, features chosen from the network had different dimensions for different cases and lacked statistical characteristics. To overcome these difficulties, we introduced an improved Fisher vector encoding^36^ for feature normalization and description. The Fisher vector was summarized in a vectorial statistic for a number of local feature descriptors by constructing a visual word dictionary obtained with Gaussian Mixture Models (GMM)^37^. Multi-scale information regarding the tumor regions was seamlessly incorporated by the description of the Fisher vector. In our study, a GMM with 64 Gaussian components was obtained based on the training data. Then, feature maps of all slices from a single case were stretched into a one-dimensional vector for each deep filter, and these were pooled into a Fisher vector representation with 64 Gaussian components. The pooled descriptors represented the first order and second order statistics of each of the 64 Gaussian components of each of the 128 deep filters, resulting in descriptors with 16k-dimensions (128 × 64 × 2). The calculations for the Fisher vector are described in the supplementary methods.

In this study, 30 patients were used to obtain the encoder of the generated CNN features for both cohorts, separately. After that, the CNN features were extracted from all of the images and encoded with a Fisher vector to obtain a 16,384-dimensional feature set for each patient. The DLR model based on multiple modalities was built and tested on the first cohort. The DLR based on a single modality was built using the second cohort and was tested on both cohorts.

### Feature selection and classification

To evaluate the ability of the CNN to recognize tumors, we assessed the tumor segmentation results of the CNN. The Dice Similar Coefficient (DSC), Positive Predictive Value (PPV) and sensitivity^38^ were calculated for the CNN tumor recognition results, with manual segmentation as the ground truth.

To select features associated with IDH1 mutation status, we included several feature selection methods in our method. Student’s t-test was applied to all of the extracted features to identify the features with significant power according to the criterion that a p-value < 0.05 indicated statistical significance. In addition, feature selection based on F-score^39^ was used for further pre-processing to remove irrelevant and redundant features.

In our study, a support vector machine (SVM) was chosen as a classifier. SVM shows good robustness and high precision, and it has been used by other studies for cancer analysis^40^. The linear kernel was chosen for the SVM in this study, and the box constraint c was set as 1.

Several indexes were calculated in this study to evaluate the predictive performance of our model. Parameters, including ROC curves, AUC, accuracy (ACC), sensitivity (SENS), specificity (SPEC), PPV, negative predictive value (NPV) and Matthew’s correlation coefficient (MCC), were calculated and presented as the prediction results. The detailed calculations for these parameters can be found in the supplementary methods.

### Dividing training data and test data

The CNN was trained using the first cohort with 119 cases and multi-modal MR images. Sixty cases from the first cohort were selected as the training data, and the remaining 59 cases were used as test data. Leave-one-out cross-validation was used for IDH1 prediction. An experiment was carried out for validation using another division of the cases. Eighty-five cases diagnosed before 2015 were chosen as the training data, and 34 cases diagnosed after 2015 were used as the test data.

### Data Availability

The datasets generated during and/or analysed during the current study are available from the corresponding author on reasonable request.

## Electronic supplementary material

Supplementary Information
