# Supplementary material for: Deep Learning based Radiomics (DLR) and its usage in noninvasive IDH1 prediction for low grade glioma
Source: Sci Rep. 2017 Jul 14;7:5467. doi: 10.1038/s41598-017-05848-2 (PMC5511238; doi:10.1038/s41598-017-05848-2)
Supplement: Supplementary file 1 — Supplementary Information [file 41598_2017_5848_MOESM1_ESM.pdf]

Supplementary Information

Submitted along with "Deep Learning based Radiomics (DLR) and its usage in noninvasive IDH1 prediction for low grade glioma", Zeju Li, Yuanyuan Wang, Jinhua Yu, Yi Guo, Wei Cao.

Supplementary Tables

Original images	CNN structures	DSC (Mean ± standard deviation)	PPV (Mean ± standard deviation)	Sensitivity (Mean ± standard deviation)
Using single modality (T2 flair)	4 conv. and 256 fc.	0.74±0.01	0.65±0.02	0.90±0.01
	4 conv. and 4096 fc.	0.74±0.01	0.65±0.02	**0.92**±0.01
	6 conv. and 256 fc.	0.75±0.01	0.66±0.02	0.91±0.01
	6 conv. and 4096 fc.	0.77±0.01	0.70±0.02	0.88±0.01
Using multiple modalities (T2 flair and T1 Contrast)	6 conv. and 4096 fc.	**0.78**±0.01	**0.81**±0.01	0.78±0.02

Table 1. Tumor segmentation results of single and multiple modal MRI images. Conv. represented the number of convolutional layers in CNN structures, and fc. represented the number of neurons in the fully connected layers in CNN structures.

The dataset used	Method	Location of feature extraction	The number of extracted features	The number of features after primary selection (t-test)	The number of features after further selection (F-score)
Second cohort	Radiomics	/	671	110	/
	DLR based on single modality	Conv. 6	16384	650	/
First cohort	DLR based on multiple modality	Conv. 6	16384	1846	494
	DLR based on multiple modality	Conv. 1	8192	38	/
	DLR based on multiple modality	Conv. 2	8192	37	/
	DLR based on multiple modality	Conv. 3	8192	1213	/
	DLR based on multiple modality	Conv. 4	16384	2010	/
	DLR based on multiple modality	Conv. 5	16384	1951	/
	DLR based on multiple modality	Fc. 7	4096	717	/
	DLR based on multiple modality	Fc. 8	4096	1099	/

Table 2. The amount of features in different cases. Conv. represented the convolutional layers and fc. represented the fully connected layers.

Layer 1	Layer 2	Layer 3	Layer 4
Conv[1]3-64	Conv3-64	Conv3-64	Max-pool
**Layer 5**	**Layer 6**	**Layer 7**	**Layer 8**
Conv3-128	Conv3-128	Conv3-128	Max-pool
**Layer 9**	**Layer 10**	**Layer 11**	**Layer 12**
FC[2]-4096	FC-4096	FC-2	Softmax

Table 3. CNN structure configurations. [1]Conv: Followed by the size of convolutional kernels and the number of filter banks. [2]FC: Followed by the number of neurons in the fully connected layers.

Supplementary Figures

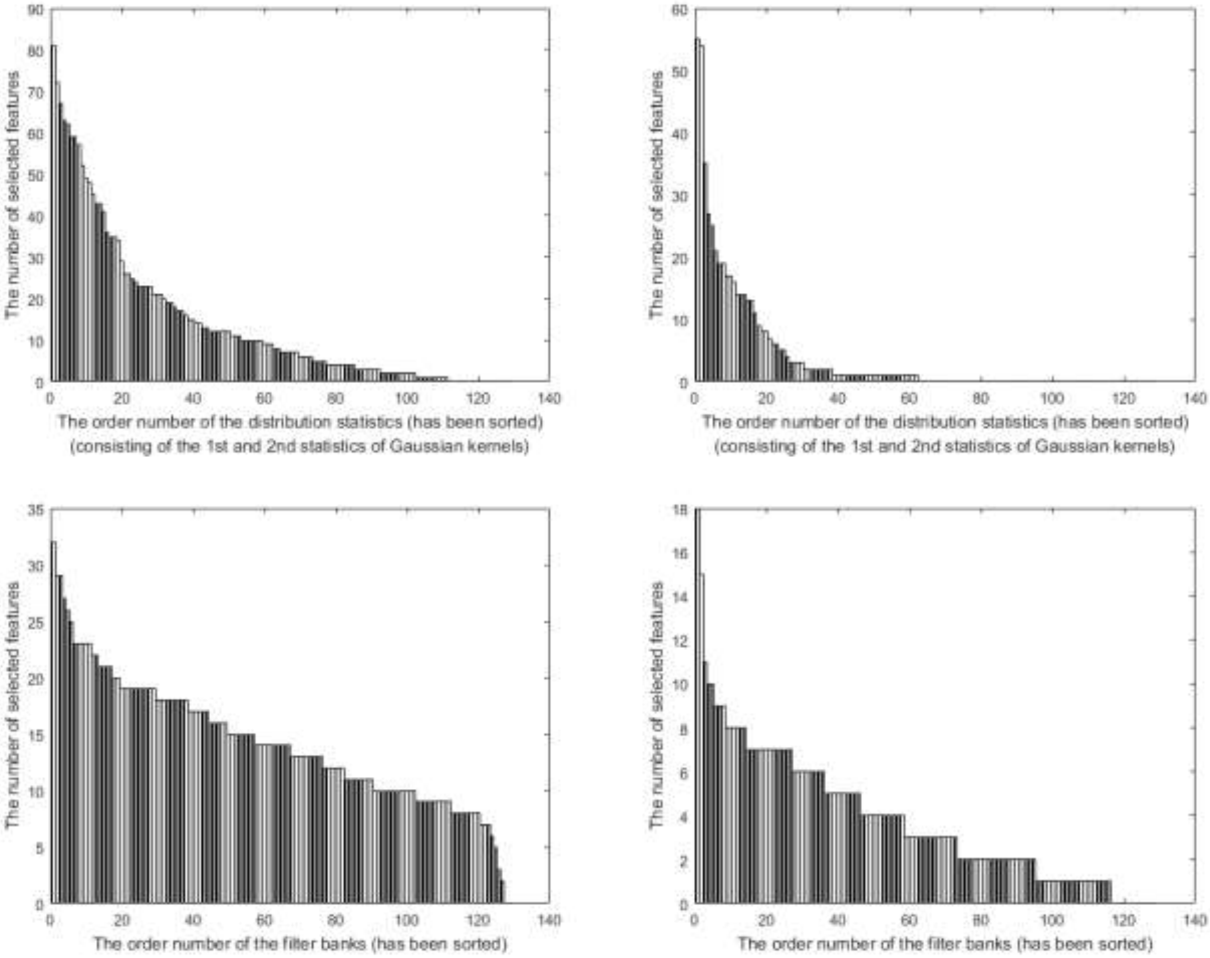

Figure 1. The number of selected features related to different Gaussian kernels and different filter banks. 1846 features are shown in the left and 494 features are shown in the right. Information related to different Gaussian kernels is shown above and information related to different filter banks is shown below. Most features were only related to a few Gaussian kernels. The significant responses to IDH1 status were observed in many filter banks.

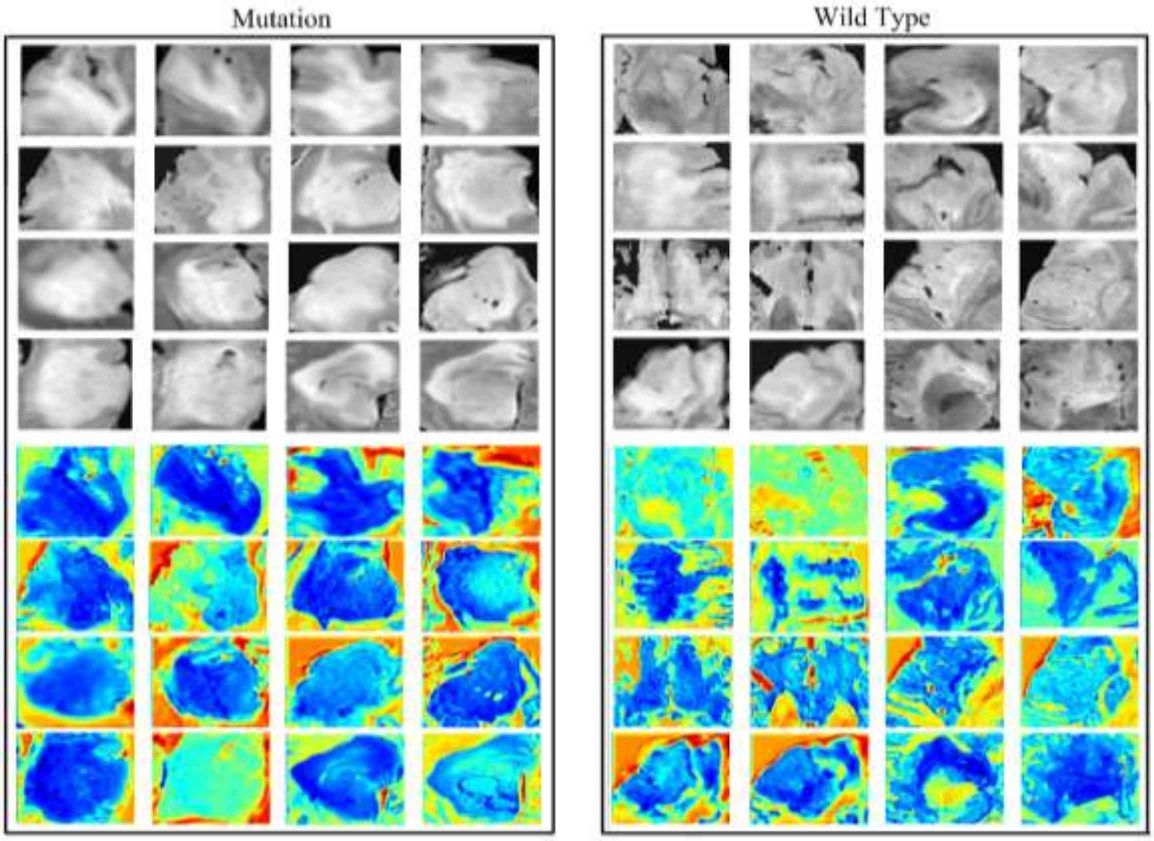

Figure 2. CNN features from the last convolutional layers. The difference between mutation and wild type IDH1 glioma of no.107 filter banks from the last convolutional layer. T2 flair images were shown above and the deep filter responses were presented below.

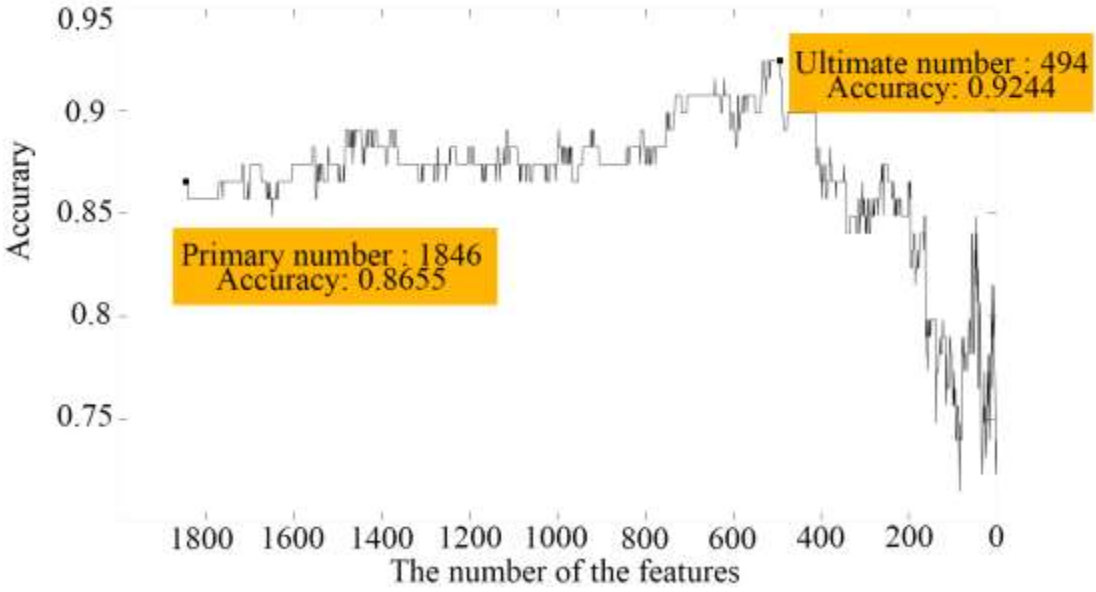

Figure 3. The improvement of prediction performance with further feature selection based on F-score.

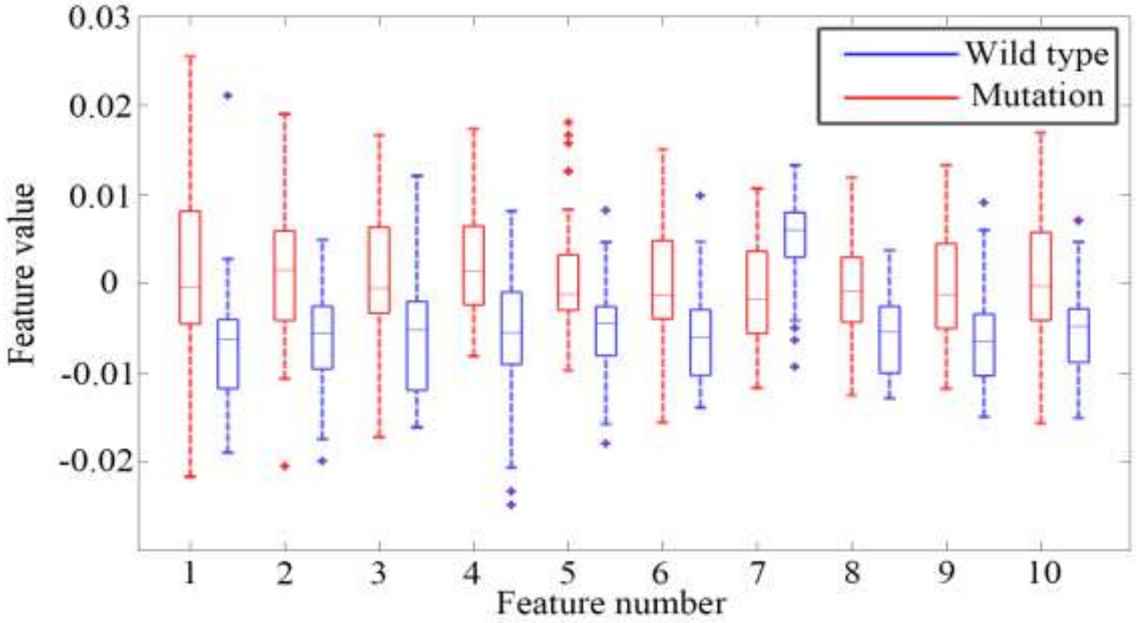

Figure 4. Boxplot of the ten most significant features. The differences between wild type and mutation were distinct in these DLR features.

Supplementary Methods

Evaluation parameters for segmentation results.

1、DSC

$$\text{DSC} = \frac{2TP}{FP + 2TP + FN}$$

2、PPV

$$\text{PPV} = \frac{TP}{TP + FP}$$

3、Sensitivity

$$\text{Sensitivity} = \frac{TP}{TP + FN}$$

where *TP*, *FP* and *FN* represent the regions of the true positive, the false positive, and the false negative, respectively.

Evaluation parameters for prediction results.

1、ACC

$$\text{ACC} = \frac{TP + TN}{FP + TP + FN + TN}$$

2、SENS

$$\text{SENS} = \frac{TP}{TP + FN}$$

3、SPEC

$$\text{SPEC} = \frac{TN}{TN + FP}$$

4、PPV

$$\text{PPV} = \frac{TP}{TP + FP}$$

5、NPV

$$\text{NPV} = \frac{TN}{TN + FN}$$

6、MCC

$$\text{MCC} = \frac{TP \times TN - FP \times FN}{\sqrt{(TP + FP)(TP + FN)(TN + FP)(TN + FN)}}$$

where *TP*, *FP*, *TN* and *FN* represent the regions of the true positive, the false positive, true negative and the false negative, respectively.

The calculation of F-score

The calculation of F-score of the *m*th feature is define as

$$F_{\text{m}} = \frac{(\overline{x_m^p} - \overline{x_m})^2 + (\overline{x_m^n} - \overline{x_m})^2}{\frac{1}{n_p - 1}\sum_{k=1}^{n_p}(x_{k,m}^p - \overline{x_m^p})^2 + \frac{1}{n_n - 1}\sum_{k=1}^{n_n}(x_{k,m}^n - \overline{x_m^n})^2}$$

where $\overline{x_m^p}$, $\overline{x_m^n}$ and $\overline{x_m}$ represent the average of *m*th feature of the positive samples, the average of *m*th feature of the negative samples and the average of *m*th feature of all the samples. n_p and n_n represent the number of positive samples and negative samples, separately. $x_{k,m}^p$ and $x_{k,m}^n$ represent the value of the *m*th feature of the *k*th sample of the positive and negative samples, separately.

The calculation of Fisher Vectors

The calculation of improved Fisher Vectors was implemented by the VLfeat toolbox in matlab. Image X gets the D dimensional feature vectors [x_1, x_2, …, x_N]. In our study, D refers to the number of filter banks and N is the total points of the feature maps. Encoder $\Theta(\mu_k, w_k . \sigma_k)$ preserves the parameters of the kth kernel of GMM. The Fisher Vector $\psi(X) = [\mathcal{G}^X_{\mu,1}, \cdots, \mathcal{G}^X_{\mu,K}, \mathcal{G}^X_{\sigma,1}, \cdots, \mathcal{G}^X_{\sigma,K}]^T$ is the combination of mean derivation vector and the covariance deviation vectors for each kernel.

$$\mathcal{G}^X_{\mu,jk} = \sum_{i=1}^{N} \mathcal{G}^X_{\mu,ijk} = \sum_{i=1}^{N} \frac{y_{ik}}{N\sqrt{w_k}} \frac{x_{ji} - \mu_{jk}}{\sigma_{jk}}$$

$$\mathcal{G}^X_{\sigma,jk} = \sum_{i=1}^{N} \mathcal{G}^X_{\sigma,ijk} = \sum_{i=1}^{N} \frac{y_{ik}}{N\sqrt{2w_k}} \left[\frac{(x_{ji} - \mu_{jk})^2}{\sigma_{jk}{}^2} - 1 \right]$$

where $j = 1, \ldots D$ is the vector dimension. y_{ik} is the posterior probability of each x_i to the kth kernel of GMM.
